# Chemosensory properties of murine nasal and cutaneous trigeminal neurons identified by viral tracing

**DOI:** 10.1186/1471-2202-7-46

**Published:** 2006-06-08

**Authors:** Nils Damann, Markus Rothermel, Barbara G Klupp, Thomas C Mettenleiter, Hanns Hatt, Christian H Wetzel

**Affiliations:** 1 Lehrstuhl für Zellphysiologie, Ruhr-Universität, Bochum, Germany; 2 Friedrich-Loeffler-Institut, Bundesforschungsinstitut für Tiergesundheit, Insel Riems, Germany; 3 International Graduate School of Neuroscience (IGSN), Ruhr-Universität, Bochum, Germany

## Abstract

**Background:**

Somatosensation of the mammalian head is mainly mediated by the trigeminal nerve that provides innervation of diverse tissues like the face skin, the conjunctiva of the eyes, blood vessels and the mucouse membranes of the oral and nasal cavities. Trigeminal perception encompasses thermosensation, touch, and pain. Trigeminal chemosensation from the nasal epithelia mainly evokes stinging, burning, or pungent sensations. *In vitro *characterization of trigeminal primary sensory neurons derives largely from analysis of complete neuronal populations prepared from sensory ganglia. Thus, functional properties of primary trigeminal afferents depending on the area of innervation remain largely unclear.

**Results:**

We established a PrV based tracing technique to identify nasal and cutaneous trigeminal neurons *in vitro*. This approach allowed analysis and comparison of identified primary afferents by means of electrophysiological and imaging measurement techniques.

Neurons were challenged with several agonists that were reported to exhibit specificity for known receptors, including TRP channels and purinergic receptors. In addition, TTX sensitivity of sodium currents and IB4 binding was investigated.

Compared with cutaneous neurons, a larger fraction of nasal trigeminal neurons showed sensitivity for menthol and capsaicin. These findings pointed to TRPM8 and TRPV1 receptor protein expression largely in nasal neurons whereas for cutaneous neurons these receptors are present only in a smaller fraction. The majority of nasal neurons lacked P2X_3 _receptor-mediated currents but showed P2X_2_-mediated responses when stimulated with ATP. Interestingly, cutaneous neurons revealed largely TTX resistant sodium currents. A significantly higher fraction of nasal and cutaneous afferents showed IB4 binding when compared to randomly chosen trigeminal neurons.

**Conclusion:**

In conclusion, the usability of PrV mediated tracing of primary afferents was demonstrated. Using this technique it could be shown that compared with neurons innervating the skin nasal trigeminal neurons reveal pronounced chemosensitivity for TRPM8 and TRPV1 channel agonists and only partially meet properties typical for nociceptors. In contrast to P2X_3 _receptors, TRPM8 and TRPV1 receptors seem to be of pronounced physiological relevance for intranasal trigeminal sensation.

## Background

Chemosensation from the mammalian nasal cavity is predominantly mediated by two independent neural systems, the olfactory and somatosensory (trigeminal) system. The primary function of the intranasal trigeminal system is to act as a sentinel of the airways [1]. It can reflexively stop inspiration to prevent inhalation of potentially life-threatening substances.

Intranasal trigeminal fibers are distributed throughout the nasal cavity and are described as intraepithelial free nerve endings arising from Aδ and C fibers of the nasopalatine and ethmoid branches of the trigeminal nerve. Most of these fibers are supposed to be nociceptive [2,3]. In many cases the ingenious defensive strategies of plants to ward off herbivorous predators are based upon the production of chemical agents such as capsaicin, isothiocyanates, and thiosulfinates that produce their behavioral effects by targeting excitatory TRP channels on primary afferent nerve fibers of the trigeminal pain pathway within the nasal and oral cavities [4,5].

TRPV1 (VR1), a member of the TRP-family, has been shown to be sensitive for capsaicin, the excitotoxic component of capsicum peppers [5,6]. TRPV1 is expressed in TGNs innervating the murine nose [7] and plays a role in perception of chemical irritants [3]. A chemosensitive function can also be attributed to the TRPM8 (CMR1) receptor channel, which can be activated by cooling compounds such as menthol, menthone, eucalyptol and icilin [8,9]. Intranasal menthol activates ethmoidal fibers [10] thereby eliciting a sensation of coldness.

Neurons of the peripheral nervous system are also endowed with ATP-sensitive receptors belonging to the P2X (ligand-gated cationic channels) and P2Y (G protein-coupled receptors) types [11,12]. Extracellular ATP can directly activate purinergic receptors on peripheral nerve terminals contributing to pain sensation (reviewed in [13]).

To date a detailed physiological *in vitro *investigation of nasal trigeminal neurons in order to solve the puzzle of their chemosensory capability has been hampered by insufficient identification in current methodological approaches. Although trigeminal fibers innervating the teeth could be traced and identified in cell culture [14], common neuronal tracers often lack adequate properties to effectively and rapidly invade the thin fiber terminals innervating the nasal epithelia.

Now we have established a pseudorabies virus mediated tracing technique for *in vitro *identification of trigeminal neurons with defined somatotopic innervation. In a double tracing approach we labeled TGNs mediating information from the murine nasal cavity (nasal TGNs) and neurons innervating the face skin (cutaneous TGNs). In patch-clamp recordings we analyzed their sensitivity to selected agonists for various receptors, including TRP channels and purinergic receptors. In order to describe nasal TGNs for the first time in more detail, the proportions of capsaicin, menthol and ATP sensitive neurons were determined and compared to cutaneous TGNs. Furthermore, TTX resistancy of sodium currents and IB4 binding, features commonly attributed to nociceptors, were analyzed.

## Results

### Tracing of nasal TGNs using pseudorabies virus

**Figure 1:**
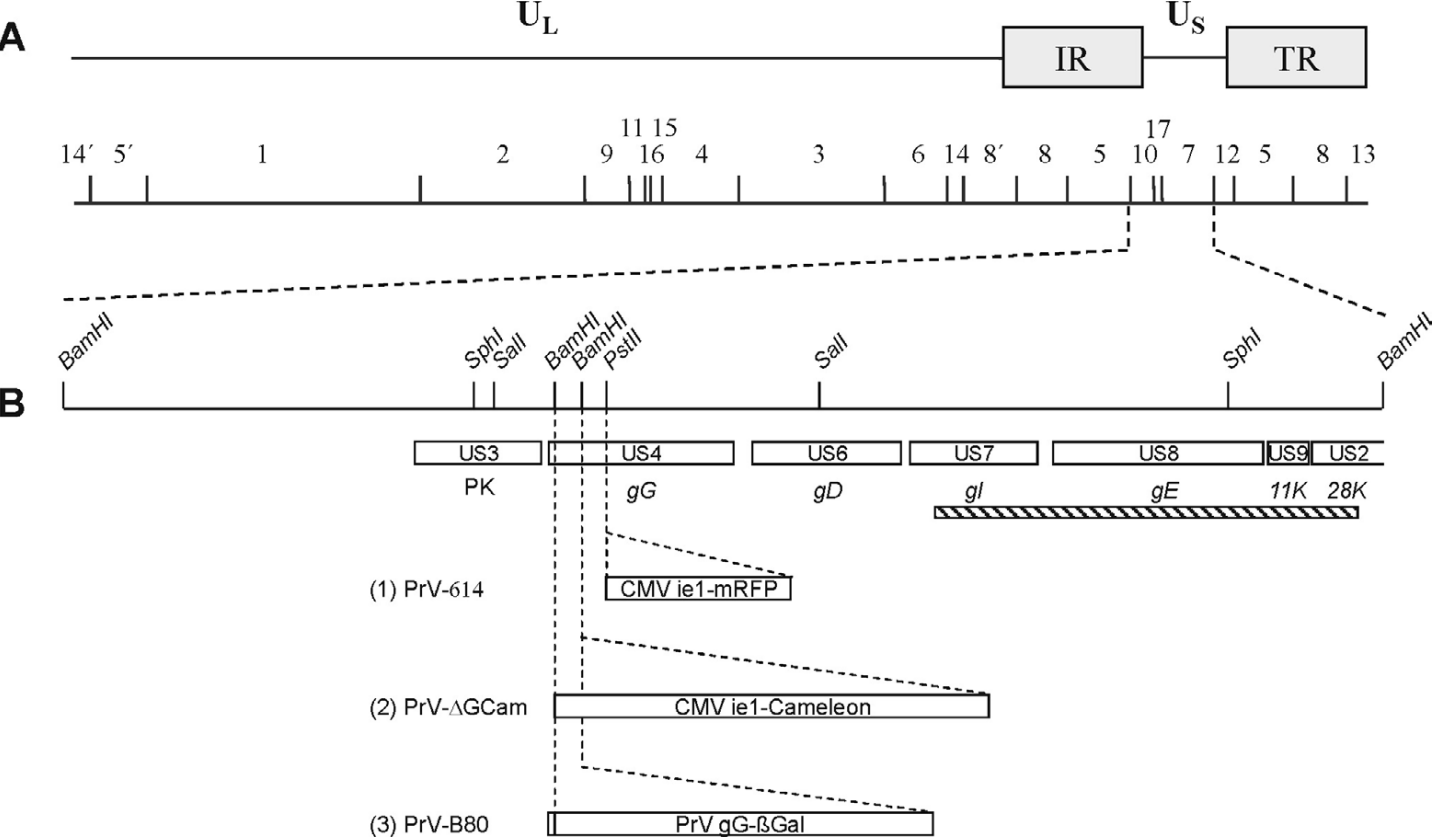
**Genomic map of the PrV-Bartha strains used in this study. **(A) shows a schematic diagram of the wildtype PrV genome with the unique long (U_L_), unique short (U_S_) and inverted repeat (IR: internal repeat; TR: terminal repeat) regions and a BamHI restriction fragment map. Fragments are numbered according to their size. In (B) the region used for insertion of marker gene cassettes is enlarged, relevant restriction sites are indicated and open reading frames are shown by rectangles. The region enlarged contains genes encoding a protein kinase (PK; US3) and glycoproteins (g)G (US4), gD (US6), gI (US7) and gE (US8), as well as the 11 K (US9) and part of the 28 K (US2) protein. The hatched box indicates the large deletion of sequences in PrV-strain Bartha compared to the laboratory strain Kaplan. PrV-614 comprises the mRFP1 under control of the CMV ie promoter/enhancer (CMV ie1) [16] inserted into the PstI site located in the gG gene. PrV-Cam expresses the Yellow Cameleon under the same promoter, substituting a short BamHI-fragment in gG, while the β-galactosidase sequences in PrV-B80 are fused to the first 8 codons of the gG open reading frame and are under control of the PrV gG gene promoter [18].

Tracing of nasal TGNs was performed using three recombinant variants of the attenuated PrV strain Bartha (Fig. 1; for review see: [15]). PrV-614 [16] carries DNA for the monomeric red fluorescent protein 1 (mRFP1), which is derived from a rapidly maturing DsRed derivative. The isogenic strain PrV-Cam comprises the gene for the Yellow Cameleon 2.1 (YC2.1). The calcium sensitive FRET effect which is described for YC2.1 by others [17] could not be shown for PrV mediated expression neither in MDBK and PK15 cell lines, nor in TGNs. However, the virally expressed protein revealed GFP like fluorescence with a yellow shifted emission spectrum. In this study the role of YC2.1 was restricted to its fluorescent capacity as marker protein. PrV-B80 [18] contains the *lacZ *gene and produces β-galactosidase as reporter enzyme.

**Figure 2:**
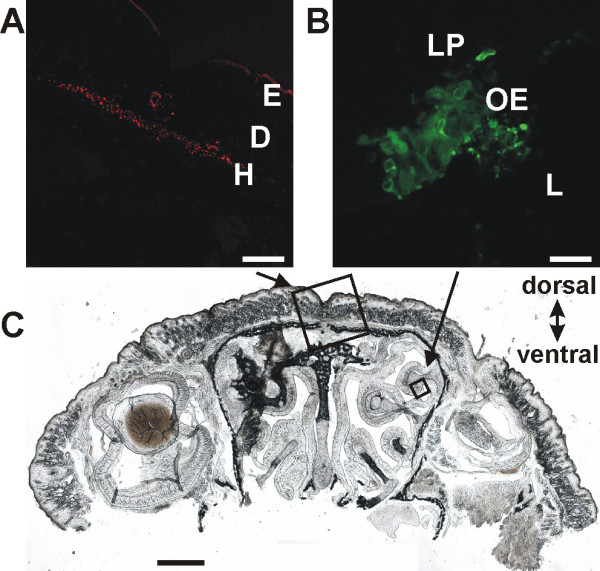
**Viral infection at inoculation sites. **(A) After intracutaneous injection of PrV-614 red fluorescent cells could be detected in the hypodermis. (B) Intranasal inoculation of PrV-Cam resulted in focal fluorescence within nasal epithelia. (C) Frontal cryosection of the murine head. E: Epidermis; D: Dermis; H: Hypodermis; LP: *Lamina propria*; OE: Olfactory epithelium; L: Lumen; Bars in A: 200 μm; B: 50 μm; c: 1 mm

Inoculating 2 μl of high titered suspension of PrV-Bartha derived viral strains into the right nasal cavity of P0 – P5 swiss (CD1) mice resulted in infection of nasal epithelia displayed by focal marker protein fluorescence (Fig. 2B). Viral protein was found exclusively within the right nasal cavity. These findings demonstrate that intranasal application of viral suspension of PrV-Bartha variants results in a robust epithelial infection, which is essential for the penetration of the thin trigeminal nerve fiber terminals. However, these observations do not rule out possible infection of deeper respiratory or oral tissues, or even the eyes, since these structures might have come into contact with nasal swabs. Infection of other tissues with trigeminal innervation than the nasal mucosa would result in loss of tracer selectivity for nasal TGNs.

**Figure 3:**
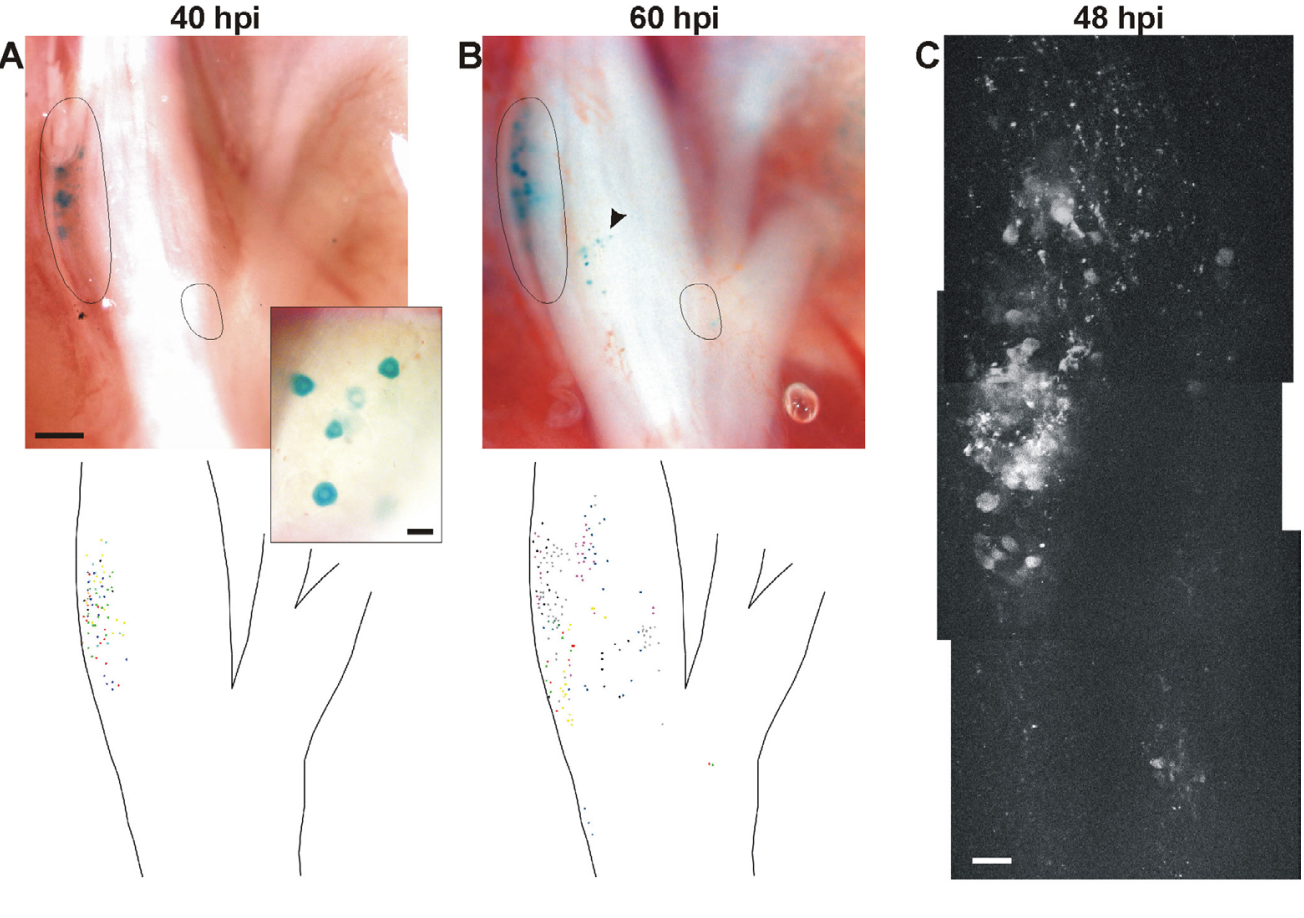
**Identification of infected cells in the TG. **(A) After inoculation of PrV-B80 to the right nostril X-gal staining identified β-gal expressing cells from a dorsal view at 40 hpi in superficial layers of the dorsomedial portion of the ipsilateral TG. (B) At 60 hpi cells appeared also in other regions (arrow head). Dashed lines indicate assumed regions with cells of ethmoidal origin. The schematic overviews compile infected cells of seven TGs each (infected cells from different animals are indicated by dots of different colors). (C) 3D multiphoton microscopy identified infected areas 48 hours after inoculating PrV-Cam exclusively in the dorsomedial portion of the ganglion. Viral invasion was mediated most likely by transport via the peripheral collaterals of neurons, since the morphology of labeled cells (A, inlay) and the speed of transportation from the nasal cavity to the trigeminal ganglion tend to exclude the involvement of glial cells. Bars in A: 200 μm, inlay 40 μm, C: 50 μm

To get information about the specificity of PrV for nasal TGNs after intranasal inoculation, the route of viral invasion was analyzed after inoculating PrV-B80 into the right nasal cavity. Whole-mount preparation of the trigeminal ganglia and subsequent X-gal staining was performed (Fig. 3). 40 hours post inoculation (40 hpi) a few (11 ± 6) β-gal expressing cells appeared in superficial dorsal layers of the ipsilateral ganglion (Fig. 3A). Preparation of the tissue 20 hours later (60 hpi) showed a more robust infection (24 ± 18 cells) of the ipsilateral ganglion (Fig. 3B) and in some cases (2/7) additional labeling at the contralateral side (data not shown). No staining could be identified at an earlier time point (20 hpi, data not shown). Stained cells at 40 hpi were restricted to the ipsilateral ganglion and appeared in the anterior dorsomedial part which is known to comprise TGNs reaching the ipsilateral nasal cavity via the ethmoid branch of the ophthalmic trigeminal division [2,19]. At 60 hpi β-gal expressing cells were scattered all over the ganglionic surface indicating ganglionic infection originating from additional tissues besides the nasal mucosa. To visualize PrV infection also in the depth of the ganglionic tissue PrV-Cam was applied to one nostril followed by detection of labeled cells using multiphoton laser-scanning microscopy at 48 hpi (Fig. 3C). In three animals tested fluorescent cells could be identified almost exclusively in the dorsomedial region of the ipsilateral ganglion.

These data suggest that within a certain time window (at about 40 hpi) a robust PrV-mediated expression of marker proteins can be achieved in TGNs that are likely to innervate the nasal mucosae. At later time points, secondary infections e.g. of the oral cavity or the eyes may account for unspecific labeling resulting in a broader distribution within the ganglionic tissue and spread to the contralateral side.

**Figure 4:**
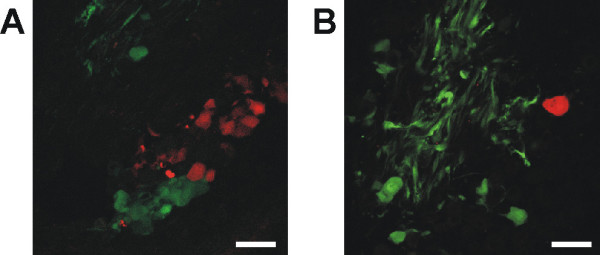
**Identification of infected cells within the trigeminal ganglia. **Two days after intracutaneous/intranasal inoculation of PrV-614/PrV-Cam both, red and green fluorescence could be detected in cryosections of the right trigeminal ganglion but never was colocalized. Bars in A and B: 50 μm

In order to identify TGNs from the subpopulation of skin innervating neurons PrV-614 was injected intracutaneously into the face skin (orbital, between the eyes). 48 hours after injection, fluorescence could be detected at the inoculation site (Fig. 2A) as well as within both ganglia. After inoculating PrV-614 (skin) and PrV-Cam (nose), red and green fluorescent neurons appeared within the right ganglion but no double labeled neurons were found indicating tracing of two independent neuronal populations (Fig.4).

### Kinetic of viral neuroinvasion

With the objective to make traced neurons accessible for patch-clamp recordings and calcium imaging measurements ganglionic tissue was taken into primary cell culture. A few days after plating, neurons exhibited cellular processes and revealed typical pseudounipolar morphology when kept under standardized culture conditions.

In order to minimize viral effects on the cellular physiology, plating of cells and *in vitro *identification of labeled neurons was intended as soon as possible after intranasal inoculation. β-Gal expression was shown for 40 hpi, indicating entry of viral DNA into the neuronal soma within the first 40 hours. To evaluate the time-point when virions reach the TG, ganglionic cells were cultured 16, 20, 24, and 48 hours after inoculating PrV-Cam into the right nostril and reporter gene expression was examined by fluorescence microscopy immediately after plating and 24 hours later. The TGN somata that were reached by the viral particles before their neuronal processes were disrupted during the preparation, should have incorporated the reporter DNA. Fluorescence, indicating viral infection, should appear immediately after plating or some hours later.

At 16 hpi no fluorescence was found immediately after plating. Even two additional days in culture did not result in detectable marker protein expression. At 20 and 24 hpi no fluorescent cells could be detected after preparation but culturing the cells for one day resulted in strong fluorescence in groups of 3 to 20 cells. Keeping cells two days *in vitro *resulted in pronounced viral spread in the culture dish due to ongoing viral replication and YC2.1-fluorescence could be detected in nearly all cell types including neurons and glial cells. Preparation after 48 hpi produced cells that were already fluorescent immediately after plating. These data imply that Bartha derived PrV strains need ~20 hours to enter the ganglionic somata of mice.

### Identification of traced TGNs in vitro

**Figure 5:**
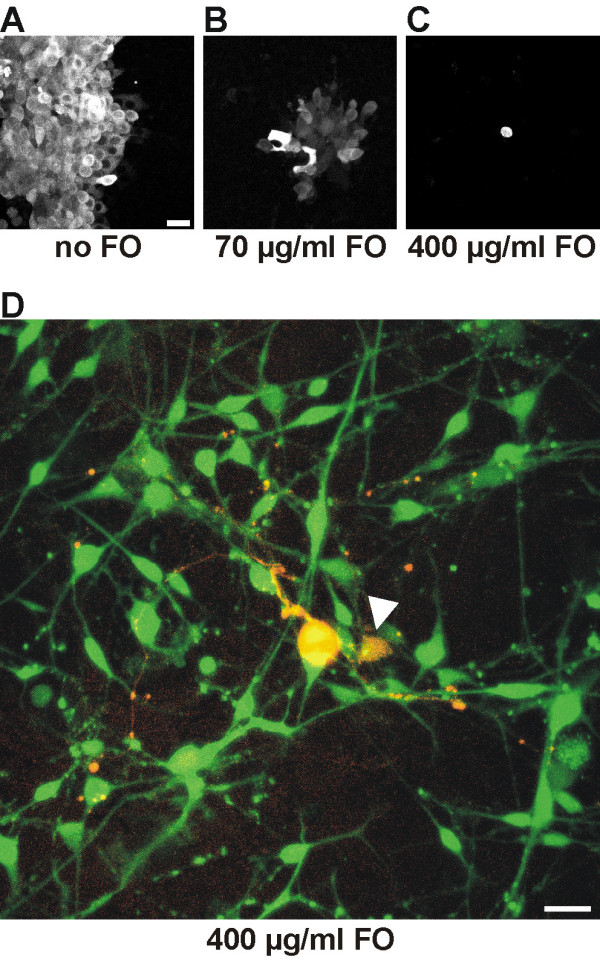
**FO inhibits viral propagation in cell culture in a dose dependent manner. **(A) Infection of MDBK monolayers with PrV-Cam without FO produced large plaques with lysed cells in the central region (picture shows a border of a plaque). (B) With 70 μg/ml FO, no plaques were formed and fluorescent cells appeared in groups of 20 – 40. (C) Presence of 400 μg/ml resulted in single fluorescent cells without viral spread to neighbouring cells. (D) Traced TGN (red) in cell culture two days after inoculating PrV-614 intranasally and one day after plating. Four hundred μg/ml FO efficiently inhibited viral spread. Cells were loaded with the calcium sensitive dye Fluo-4 (green). Red and green fluorescences were merged in this figure. Arrowhead indicates an infected glial cell. Bars in A: 20 μm, D: 30 μm

Ongoing viral spread after plating of dissociated trigeminal cells at 20 hpi ruled out identification of traced neurons. After one day *in vitro *most neurons were lysed due to viral propagation and cytopathic effects. To prevent viral replication in cell culture, the pyrophosphate analogue foscarnet (phosphonoformic acid, FO) a direct non-competitive inhibitor of viral DNA-polymerase was added to the culture medium. FO is known to inhibit the propagation of several types of viruses, including HSV-1 and CMV. A plaque assay using MDBK monolayers was used to determine the FO concentration that allowed reliable inhibition of PrV replication. A concentration of 400 μg/ml culture medium proved to be sufficient to inhibit viral spread for at least three days (Fig. 5 A-C). This concentration turned out to be appropriate for preventing secondary infection in trigeminal cell culture as well (Fig. 5D), allowing reliable identification of traced cells after nasal and cutaneous inoculation. For identification of traced nasal and cutaneous TGNs in calcium imaging experiments PrV-614 was used because the mRFP1 emission spectrum is clearly distinguishable from that of the calcium sensitive dye Fluo-4. In phase contrast light microscopy, the general appearance of traced cultured neuronal cell bodies did not differ from non-infected neurons, therefore traced cells could only be identified using fluorescence microscopy. Infected and markerprotein expressing cells within FO treated cultures showed predominantly typical TGN morphology with spherical and pseudounipolar shape and long processes already at day one *in vitro*. Only a few cells had a flattened soma and thus were classified as glial cells. This classification was confirmed by calcium imaging experiments: Treating the cells with 45 mM KCl induced robust calcium signals only in cells showing neuron-like morphology. mRFP1 expression could be observed in 3 to 10 neurons per cultured ganglion two days after plating. The number of traced neurons in culture declined on day 3 and 4 indicating apoptotic processes and cell lysis.

### Biophysical properties of infected neurons

Establishment of this live cell tracing technique now allowed investigation of physiological properties of identified trigeminal neurons *in vitro*. To rule out possible influence of viral infection on cellular physiology, we performed detailed analysis using whole-cell patch-clamp recordings of cultured TGNs. Control experiments were designed to compare nasal TGNs with non-infected control cells (control TGNs), and *in vitro *infected neurons (*iv infected *TGNs). The latter were infected with PrV-suspension directly applied to the culture dish (multiplicity of infection: ~100 PFU/cell). Cultivation for one day before *in vitro *infection allowed growth of cellular processes that might be crucial for viral invasion [21,22].

**Figure 6:**
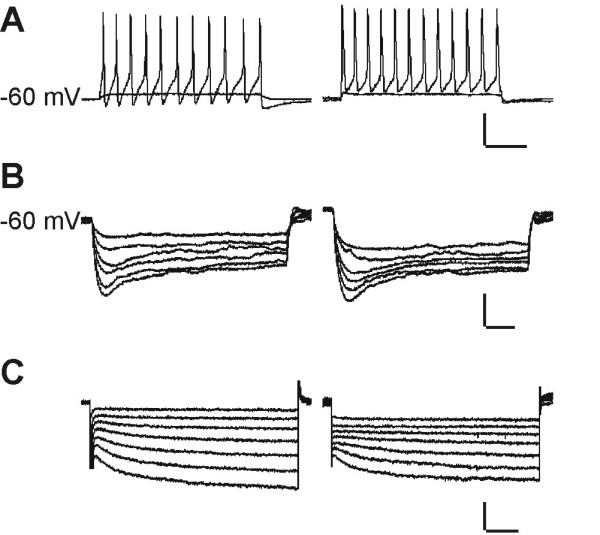
**Exemplary traces from patch-clamp recordings. **Traced TGNs (left) could not be distinguished from uninfected control neurons (right). (A) Current injection induced repetitive action potentials. (B) I_h _channel activation in current clamp mode. (C) I_h _current in voltage clamp. Bars in A: 40 mV/100 ms; B: 20 mV/300 ms; C: 200 pA/300 ms

Electrophysiological parameters were recorded from cells one to four days *in vitro *and revealed no significant differences and, therefore could be combined for statistical analysis (summarized in Tab.1). Finally, none of the criteria tested gained significance (for p < 0.05; unpaired Student's t-test) for changes in electrophysiological properties due to viral infection or FO treatment (data not shown) of cultured TGNs. Exemplary recordings from traced and uninfected control cells are illustrated in Fig. 6. Detailed comparison of nasal TGNs with cutaneous TGNs did not show any significant differences in their electrophysiological characteristics.

**Figure 7:**
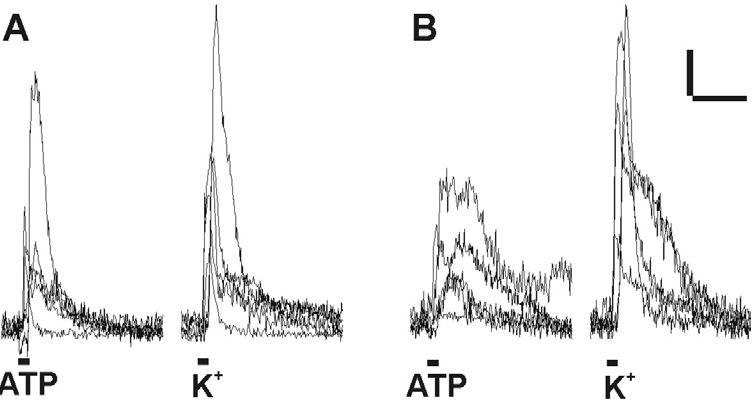
**Exemplary traces from calcium imaging measurements. **(A) Control and (B) nasal TGNs showed robust increase of intracellular calcium due to ATP stimulation or depolarization by 45 mM extracellular KCl. Bars: Δ Emission = 25, 30 s

Furthermore, the calcium imaging technique was used to assess possible alterations of the excitability when neurons were depolarized by an extracellular high potassium containing solution (45 mM KCl). In all traced cells which were identified as neurons by their morphology (6/6), depolarization induced a strong calcium influx (Fig. 7).

Traced infected cells were also compared with non-infected neurons for their sensitivity for ATP. Most TGNs are known to express ATP sensitive purinoceptors (P2X), whose different subtypes form multimeric cation selective ion channels (for review see [20]). Five of 6 traced neurons could be activated by ATP and showed a strong increase in calcium dependent fluorescence (Fig. 7). In line with the characterization of traced trigeminal neurons, analysis of ATP mediated responses will be described in more detail below.

Our findings demonstrated that traced neurons can be identified *in vitro *and retained physiological properties under FO treatment for 1 to 4 days in culture which is necessary for further analysis of sensory features.

### Sensitivity of nasal/cutaneous TGNs for menthol and capsaicin

**Figure 8:**
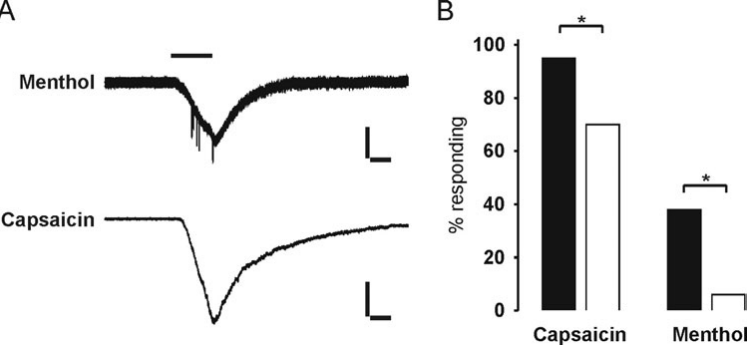
**Response of neurons to capsaicin (1 μM) and menthol (1 mM). **(A) Exemplary traces from patch-clamp recordings. Bars 1 s, 100 pA (menthol trace); 1 s, 500 pA (capsaicin trace) (B) Fractions of neurons responding to capsaicin and menthol. Black bars: nasal TGNs; White bars: cutaneous TGNs.

Nociceptors are characterized, in part, by their sensitivity to capsaicin. Traced TGNs were analysed in patch-clamp measurements for their sensitivity to this agonist. Furthermore, sensitivity for menthol was investigated. In general this agonist activates neurons that were shown to lack characteristics of nociceptors but could be activated by non-noxious cooling. To date TRPV1 and TRPM8 are the only capsaicin- and menthol-sensitive proteins identified, respectively [6,8,9]. A very large fraction of nasal TGNs (19/20) could be stimulated by capsaicin in patch-clamp recordings whereas significantly fewer cutaneous neurons were sensitive to this vanilloid compound (14/20, n = 3 experiments; p = 0.037, unpaired Student's t-test) (Fig. 8). In patch-clamp recordings significantly more nasal (6/16; 38%) than cutaneous TGNs (1/18; 6%) were sensitive to menthol (p = 0.021). Exemplary inward currents are shown in Fig. 8A. The subpopulation of nasal TGNs comprised more menthol and capsaicin sensitive cells than the group of cutaneous TGNs.

### TTX-sensitivity of sodium currents

**Figure 9:**
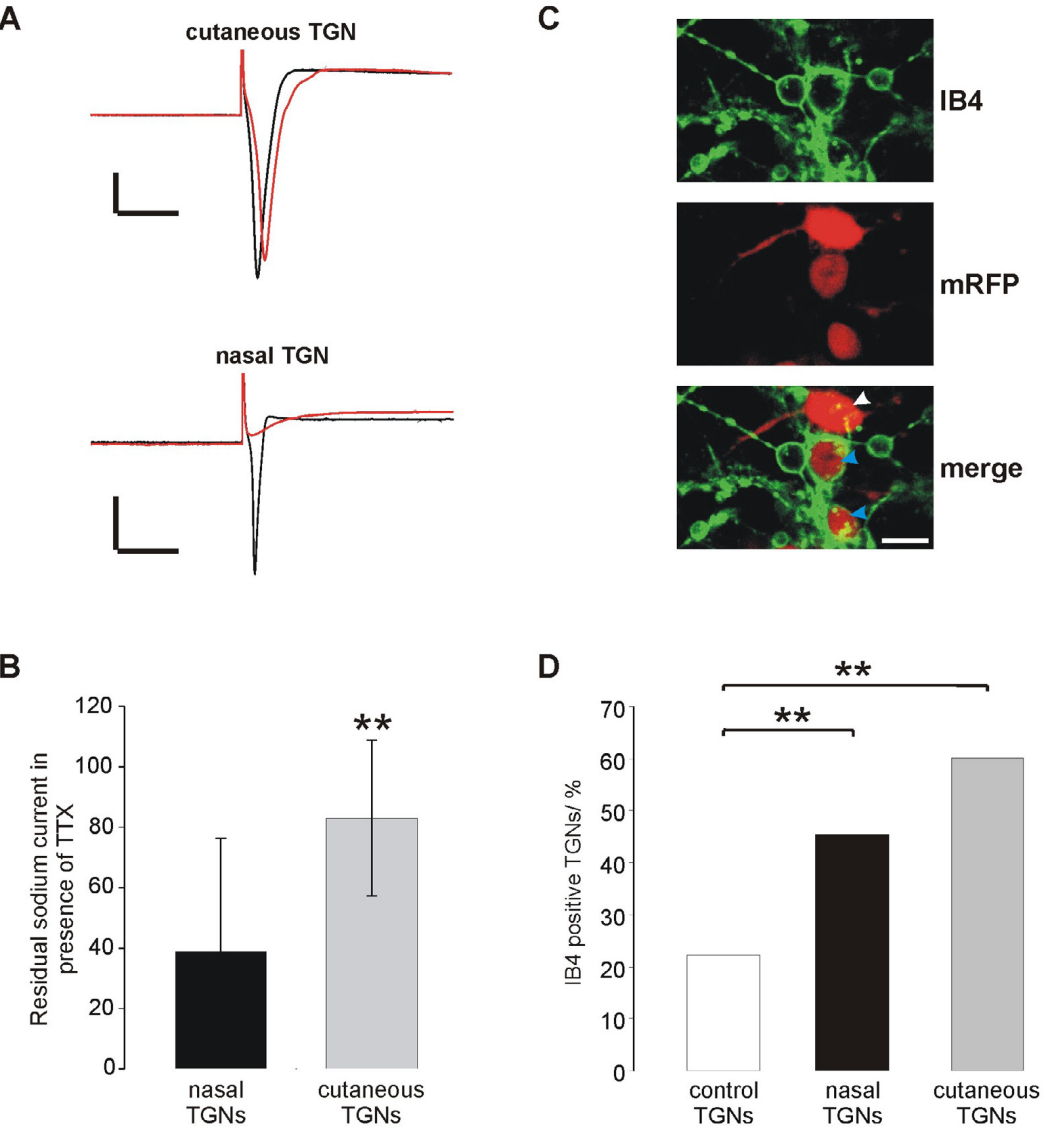
**Analysis of traced TGNs for TTX-sensitivity of sodium currents and IB4 binding. **(A) Exemplary sodium inward currents of nasal and cutaneous TGNs without (black) and in presence of 500 nM TTX (red). Vertical bars: 2 nA; Horizontal bars 10 ms (B) Mean amplitude of sodium inward currents in presence of TTX. Sodium currents were normalized to currents without TTX (= 100%). Error bars indicate standard deviation. (C) IB4 staining of TGN cell culture following *in vivo *infection (intranasal inoculation of PrV-614). White arrowhead: IB4 negative traced neuron, Blue arrowheads: IB4 positive traced neurons, Bar: 10 μm (D) Fractions of IB4 binding neurons within the groups of control (n = 72), nasal (n = 19), and cutaneous (n = 36) TGNs.

Fast sodium currents in DRGs/TGNs can be differentiated by their sensitivity to tetrodotoxin (TTX) [23-25]. Fig. 9A shows selected patch-clamp traces from nasal TGNs and cutaneous TGNs. Most neurons revealed partial TTX mediated inhibition of sodium currents but calculation of the mean values revealed that traced sodium currents of cutaneous neurons showed significantly more TTX-resistancy than sodium currents of nasal TGNs (nasal TGNs: 38.8 ± 37.5% cutaneous TGNs: 83 ± 25.8%, p = 0.001, unpaired Student's t-test, two independent experiments) (Fig. 9 A+B).

### IB4 binding

The isolectin B4 from *Griffonia simplicifolia *type one (IB4) is assumed to bind to a subpopulation of small sized DRG nociceptors [26]. In the TGN cell culture binding of the fluorescence labeled IB4 revealed a spherical green ring at the soma (Fig. 9C) of 22% of all neurons in cell culture. Neuronal processes are labeled as well. Fig. 9C illustrates IB4 binding (green) in a TGN cell culture that was *in vitro *infected with PrV-614, showing IB4 binding and non-binding infected neurons. In comparison with control TGNs in two independent experiments a significantly higher percentage of nasal TGNs (20/44; 45%; p < 0.01, χ^2^-test) and cutaneous TGNs (53/88; 60%; p < 0.01) were positive for IB4 (Fig. 9D).

### Purine-induced responses of traced TGNs

**Figure 10:**
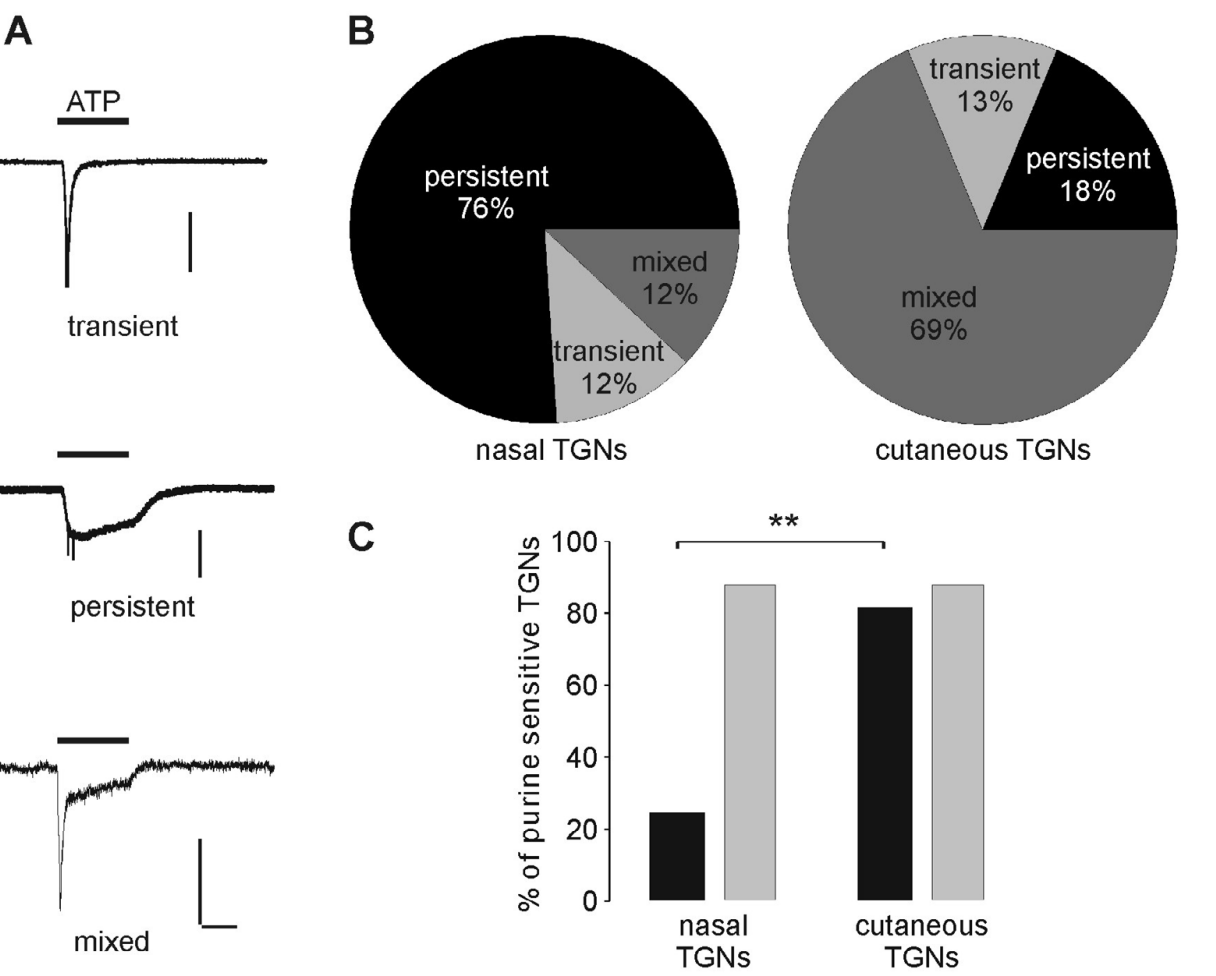
**Purines induce different TGN responses. **(A) Application of ATP (30 μM) revealed three types of inward currents in patch-clamp measurements. Horizontal Bar: 1 s; Vertical bars in from above: 250 pA, 250 pA, 100 pA (B) Current characteristics of nasal (n = 25) and cutaneous (n = 16) TGNs due to purinergic stimulation. (C) Transient responses within the groups 'transient' and 'mixed': black bars; Persistent responses within the groups 'persistent' and 'mixed': grey bars. Both neuronal subpopulations equally revealed the persistent response but cutaneous TGNs showed significantly more transient currents.

When measured by patch-clamp recordings, populations of neurons could be differentiated by three different kinetics in response to ATP (Fig. 10A): (i) slowly activating inward currents that weakly desensitized during 4 s application of ATP (30 μM) ('persistent current'); (ii) neurons showing phasic inward currents, which were characterized by a rapid current onset, higher amplitude, and fast desensitization. These currents desensitized almost completely within the application time ('transient current'). (iii) the third group of neurons exhibited ATP-induced responses consisting of two components: a fast desensitizing inward current followed by a second component showing a sustained response in presence of ATP ('mixed current') (compare [27]).

It is already known that various P2X receptors differ in their sensitivity to the ATP analogue α,β-methylene ATP (α,β-meATP). Whereas α,β-meATP activates homomeric P2X_3 _and heteromultimeric P2X_2/3 _receptors in very low concentrations (the EC_50 _is 1 μM and 9 μM, respectively) [28,29], homomeric P2X_2 _receptors can be activated by α,β-meATP only at concentrations higher than 100 μM [20]. In this study, application of 10 μM α,β-meATP also exclusively activated the transient current, supposedly induced by the P2X_3 _receptor subtype [27].

In patch-clamp measurements 81% (25/31) of nasal TGNs and 84% (16/19) of cutaneous TGNs responded to purines (ATP or α,β-meATP) (n = 4 experiments). Further analysis of the inward currents of purine sensitive neurons revealed that a large fraction of nasal TGNs (76%; 19/25) responded with ATP inducible persistent currents (Fig. 10B), whereas only 18% (3/17) of cutaneous TGNs showed currents with persistent kinetics resulting most probably from exclusive P2X_2 _receptor expression. ATP-induced currents with transient P2X_3_-like kinetics were evoked in only 24% (6/25) of the nasal TGNs, but in 82% (13/16) of the cutaneous TGNs (Fig. 10C). These findings indicate the presence of purine-induced P2X_3 _currents as found in nociceptors in a large population within the group of cutaneous TGNs and only in a small population of nasal TGNs.

## Discussion

Primary sensory neurons of the trigeminal nerve (V, cranial) provide most of the somatosensory information of the mammalian head. They mediate mechanical, thermal and chemosensory information from many tissues including the meninges, the cornea and conjunctiva of the eyes, the face skin and the mucous membranes of the oral and nasal cavities. Thereby single trigeminal neurons (TGNs) are specialized for different somatosensory modalities [30] and mediate sensations from selective areas of the head (receptive fields).

Differential sensory properties of primary afferents depending on their area of innervation and somatosensory function remain largely unclear. Characterizations of traced, somatotopically defined sensory neurons were mostly performed using immunohistochemical approaches in fixed tissue of trigeminal ganglia. Characterization of the physiology of living trigeminal or spinal primary sensory neurons derives largely from *in vitro *analysis of complete neuronal populations prepared from sensory ganglia and does not consider the area of peripheral innervation. Electrophysiological analysis in cell culture of trigeminal fibers innervating the teeth could be performed using DiI tracer [14]. However, common neuronal tracers lack adequate properties to effectively and rapidly invade the thin trigeminal fiber terminals innervating the nasal epithelia impeding *in vitro *identification of nasal TGNs.

In this study, a PrV based tracing technique was established to identify nasal trigeminal neurons *in vitro*. Neurons were challenged with several agonists that were reported to exhibit specificity for known receptors, including TRP channels and purinergic receptors. Analyzing the sensitivity of identified nasal trigeminal neurons for selected chemical stimuli aimed to improve our understanding of mechanisms underlying nasal trigeminal somatosensation.

The neurovirulent capacity of PrV-Bartha variants have been exploited in numerous studies for tract tracing after intracerebral [31-33] or peripheral [34-40] injection (for review see [41]). For tracing trigeminal afferents in this study, three variants of PrV-Bartha have been used, PrV-B80, PrV-Cam and PrV-614. These strains express marker proteins in early stages of infection, allowing identification of traced cells shortly after viral particles have entered the neuronal soma. This tracing approach revealed no obvious differences in kinetics of viral progression (*in vitro *and *in vivo*) between the Bartha strains that were used. In accordance with these observations, it has previously been shown that virus replication of PrV-614 does not differ from that of the parental PrV-Bartha strain at least on PK15 monolayers [16]. Analysis of neuroinvasiveness and neurotropism has shown similar, although not identical characteristics of strains that are isogenic to PrV-Bartha and carry the reporter gene under control of the P_hCMV _promoter/enhancer within the gG locus [16]. Therefore, viral propagation of the used Bartha variants should be similar.

In order to analyze specificity of PrV-Bartha mediated tracing for nasal trigeminal neurons after intranasal inoculation of high titered viral suspension, virus driven marker protein was localized in whole mount preparations of the trigeminal ganglia. From a topographic aspect, there is a general agreement that the somata of the mandibular nerve occupy the posterolateral portion of the ganglion, the cell bodies of the ophthalmic branches are located anteromedially, and the perikarya of the maxillary branches are interposed in-between [2,19,42,43]. After intranasal inoculation of PrV-B80/PrV-Cam, infected trigeminal somata were identified in the anterior dorsomedial part of the TG (40 hpi, β-gal staining, 48 hpi multiphoton microscopy). This localization is typical for ethmoidal neurons comprising most of the nasal trigeminal fibers [2,42,44]. Strictly ipsilateral localization of nasally traced TGNs is in agreement with findings by Anton and Peppel [2] and argues for tracer selectivity at least in early stages of infection. In later stages viral markers appeared more scattered on the ipsilateral ganglion and emerged on the contralateral side as well. Both might be due to secondary infections of tissues other than the nasal mucosa. These findings underlined the impact of the incubation period after inoculation to maintain selectivity for tracing trigeminal neurons innervating the nose.

In order to identify traced neurons *in vitro*, TGNs were dissociated and plated. Analysis of time-dependent marker protein expression in nasally traced TGNs in cell culture indicated a critical time window between 20 and 24 hpi for preparation and cultivation of cells to achieve selective tracing of nasal neurons. The time of incubation was not elongated to minimize virus induced cytopathic effects. Reliable identification of cultured traced neurons *in vitro *required prevention of viral spread within the culture dish. FO mediated pharmacological inhibition of viral replication was first established in a plaque assay on monolayers of MDBK cells and turned out to effectively prevent viral spread in cell culture of TG tissue as well. Furthermore, inhibition of viral replication in early stages of infection should minimize viral influence on the neuronal physiology. In summary, FO treatment of cell cultures allowed identification of virally labeled neurons and was crucial for the correct identification of traced cells. FO was shown to have no influence on the cellular physiology.

Intracutaneous PrV injection identified trigeminal neurons innervating the skin of the face. Double tracing of nasal and cutaneous TGNs allowed direct comparison of both neuronal populations for selected cellular features. Single TGNs might have large receptive fields and may target different tissues in the periphery at the same time. It has already been reported for nasally traced TGNs that they hold peripheral collaterals which have contact with the olfactory bulb [19]. A prerequisite for the present study was strict separation of the receptive fields of nasally traced and cutaneous neurons. Although the orbital skin is in close proximity to the nose and nasal and cutaneous trigeminal neurons have processes within the same trigeminal division (the ophthalmic nerve), the double tracing approach showed that none of the fluorescent neurons within the TG exhibited double fluorescence. This indicates the presence of two independent populations of neurons involved in mediating sensations from the nose or the orbital face skin. In order to investigate the general capability of both viral strains to co-infect individual TGNs, we performed simultaneous *in vitro *infection of trigeminal cell cultures with PrV-614 and PrV-Cam. We found that 80% (33/41) of neurons revealed virus induced fluorescence. 20% (8/41) of all neurons showed exclusively green and 22% (9/41) showed red fluorescence. 39% (16/41) of neurons revealed double fluorescence, indicating that both viral strains are able to co-infect individual TGNs at the same time.

Unaltered biophysical properties of PrV-infected TGNs is a prerequisite to characterize the physiology of virally traced neurons *in vitro*. Despite the inhibition of viral replication by use of FO, influences of PrV on neuronal physiology due to replication independent processes could not be ruled out. E.g. *UL41 *encodes a tegument protein with RNase activity, namely vhs, that degrades host mRNA in a manner similar to its HSV-1-encoded homolog [45,46]. vhs is responsible for the virion host shut-off of cellular protein synthesis. Tegument proteins like vhs play important roles during viral entry and virion morphogenesis [45]. Following fusion of the viral envelope with the plasma membrane, the tegument proteins enter the cell with the capsid and assist with host-cell takeover. These events occur before any viral proteins are synthesized.

To investigate the influence of viral infection on the cellular physiology, detailed analysis of biophysical properties using whole-cell patch-clamp recordings of cultured TGNs has been performed. Control experiments have been designed to compare traced TGNs with non-infected control TGNs, and *in vitro *infected neurons. Biophysical parameters of cells up to four days *in vitro *were recorded with electrophysiological methods and revealed no significant differences between the groups of traced, *in vitro *infected and non-infected neurons. In contrast to other publications that reported HSV induced changes in neuronal excitability of cultured DRG neurons [47,48], PrV-Bartha infection did not lead to changed electrophysiological properties. Using brain slice preparations after transsynaptic tracing with PrV-Bartha recombinants, others performed electrophysiological experiments on neurons related to the parasympathetic circuit of the heart [39,49] or stomach [50], or on retinorecipient neurons of the suprachiasmatic nucleus [36]. They demonstrated that excitability of infected cells was not different from non-infected neurons, which is consistent with our findings. Detailed comparison of nasal with cutaneous TGNs did also not show any significant differences in their electrophysiological characteristics. In addition, in calcium imaging measurements traced and non-infected neurons revealed intracellular calcium transients when 30 μM ATP or 45 mM extracellular KCl was applied. In conclusion, data from this work and the literature indicate that PrV-Bartha mediated live-cell tracing can be performed, allowing analysis of the physiology of identified neurons *in vitro*.

The primary function of the intranasal trigeminal system is to detect potentially life-threatening substances within the inhaled air. Noxious stimuli within the nasal cavity elicit pain, described as a stinging, burning, or pungent sensation. Cutaneous sensations have to be less sensitive to air born compounds but pain perception is relevant to detect and prevent potentially damaging stimuli e.g. strong mechanical forces. Nociceptor profiles derive largely from analysis of fibers that innervate the skin, but very different features characterize nociceptors in other tissues [51]. For example, although corneal afferents can be activated by capsaicin and sensitized by inflammatory mediators, pain is normally produced by innocuous tactile stimulation. In teeth, almost any stimulus produces pain. Visceral pain is unique in that there are no first (fast) and second (slow) components; instead pain is often poorly localized, deep and dull [52]. Therefore, the sensation of pain might have different relevance depending on the tissue of sensory innervation. Nociceptors are poorly characterized in correspondence with the tissue of innervation.

In this study, the viral tracing technique was combined with *in vitro *techniques like patch-clamp recordings and calcium imaging measurements to analyze nasal and cutaneous trigeminal neurons. A drawback of working with cultured cells might be that neurons are not kept in their natural environment. The plating procedure stresses cells and disrupts neuronal processes containing receptor proteins but measurements from single trigeminal neurons in the ganglia can hardly be achieved in the *in vivo *situation. Long-term maintenance of DRG neurons in culture (several days to weeks) and presence of NGF has been shown to significantly alter phenotype of neurons, particularly with respect to TTX and capsaicin sensitivity [53-55]. In this study, neurons were maintained for the shortest period possible and all recordings were made within 24 h after preparation (except for the analysis of the electrophysiological properties). Cells were cultured without addition of NGF to the growth medium.

Nociceptors are classically defined by molecular markers that originate from histochemical studies or are identified by activation thresholds for pain producing stimuli. A chemosensory hallmark of nociceptors is the pronounced capsaicin sensitivity. In the study at hand, patch-clamp recordings revealed that compared with trigeminal neurons innervating the skin, a higher proportion of nasal TGNs possess sensitivity for the TRPV1 specific agonist capsaicin. In summary, the large fraction of capsaicin sensitive nasal TGNs allows speculation about a specific role of TRPV1 receptors in nasal trigeminal afferents.

Pronounced capsaicin sensitivity and IB4 binding is attributed to nociceptors and has been described for nasal TGNs in this study. Further investigation in comparison with cutaneous TGNs revealed rather non-nociceptor characteristics of nasal TGNs: (i) Traced nasal neurons showed significantly less TTX-resistant sodium currents than cutaneous TGNs (nasal TGNs: 38.8 ± 37.5% cutaneous TGNs: 83 ± 25.8%, p = 0.001). Several types of sodium currents resistant to micromolar concentrations of TTX have been identified in DRG neurons, and some evidence suggest that these channels play an important role in the transmission of nociceptive information [23-25]. (ii) A large fraction of nasal TGNs, challenged with ATP, showed sustained inward currents typical for homomeric P2X_2 _receptors [28]. In contrast responses with mixed P2X_2_/P2X_3 _kinetics predominated in cutaneous TGNs. Purinergic P2X_3 _receptors are expressed on primary afferent small diameter neurons of the PNS and seem to be of particular importance in pain sensation [56].

In conclusion, a large fraction of nasal TGNs showed chemosensitivity for menthol and capsaicin. This suggests that nasal TGNs might also exhibit thermosensory capacities because the corresponding TRP channels are described for their pronounced thermosensitivity. Intranasal detection of temperature of the inhaled air should be fundamental for the survival of a mammalian organism. With regard to the features under investigation, nasal TGNs revealed fewer characteristics of classically defined nociceptors. Instead nasal TGNs revealed purine induced P2X_2_-like currents. Interestingly, modulation of P2X_2 _receptor mediated currents by the trigeminal agonist benzaldehyde and chemically related substances has been recently described [27]. A high fraction of nasal TGNs showing sustained ATP currents strengthens the hypothesis that these P2X_2 _specific effects could be relevant for trigeminal chemoperception.

## Conclusion

Using recombinant PrV strains rapid labeling of nasal and cutaneous trigeminal neurons of young mice was performed. Living cells could be identified *in vitro *by PrV-driven marker protein expression. In contrast to PrV-infected TGNs grown in conventional culture medium, cells grown in FO-supplemented medium retained functionality for several days. Under these conditions all tested physiological parameters were essentially identical to non-infected cultured TGNs. Using this approach nasal TGNs were analyzed in comparison to cutaneous TGNs for specific sensory aspects. Analysis and comparison of traced nasal and cutaneous neuronal populations for common nociceptor markers revealed clear differences in the respective fractions of neurons. Further characterization of PrV labeled somatosensory neurons after inoculation into other target tissues will further contribute to the understanding of differential somatosensation. Our findings suggest that in contrast to P2X_3 _receptors, TRPM8 and TRPV1 receptors have pronounced physiological relevance for intranasal trigeminal sensation.

## Methods

### Animals

The study was conducted using 0–5 days-old Swiss mice (CD1, Charles River). The animals were caged, with water and commercial food *ad libidum*. All animal experiments were carried out in accordance with the European Union Community Council guidelines. The virus-infected animals were kept separated, and biosafety level 2 precautions were applied.

### Construction of PrV-Cam

The YC2.1 under control of the CMV promoter/enhancer complex was excised from plasmid pcDNA3-YC2.1 [17] and inserted into the cloned genomic SphI-fragment of PrV-strain Kaplan [57] thereby substituting a 196 bp BamHI fragment in the nonessential gG gene (see Fig. 1). After transfection of genomic PrV-Bartha DNA with the resulting plasmid green fluorescent plaques were picked and purified. DNA of single plaque isolates was tested by Southern blot analysis for correct recombination (data not shown) and absence of gG but presence of YC2.1 expression was tested in western blot analysis of infected cell lysates (data not shown). One plaque isolate was chosen for further analysis and named as PrV-Cam.

### Virus and inoculation procedure

The PrV strains PrV-614, PrV-Cam and PrV-B80 were propagated in monolayers of porcine kidney (PK15) cells, and assayed using a standard plaque assay. Viral stocks containing 5 × 10^8 ^PFU/ml were aliquoted in 100 μl volumes and stored at -80°C. At the times of inoculation, viral aliquots were removed from the freezer and kept on ice until used.

For intranasal inoculation, mice were placed on their backs and a 2 μl drop of viral suspension (~10^6 ^PFU) was applied to the right nostril. Viral suspension was soaked up into the nasal cavity by regular breathing of the non-anesthetized pups. Animals were kept in that position for a few minutes to ensure contact of the *inoculum *with the nasal epithelium. For tracing nerve fibers from the facial skin, animals were cooled down on ice for some seconds and 1 μl of viral suspension was injected intracutaneously into the orbital skin between the eyes.

Experimental *in vitro *infection of neuronal cultures was performed 24 h after plating at a multiplicity of infection of 100 PFU/cell, using a 1 h incubation at 37°C, followed by two changes with growth medium containing 10% serum to remove unabsorbed virus. Infected and uninfected cultures were incubated in growth medium containing foscarnet (FO, Fluka) (400 μg/ml) at 37°C for up to 4 days before experiments were performed.

### Primary culture of trigeminal sensory neurons

The primary cell culture of trigeminal neurons has been described previously [27] and was only slightly altered. For each series of experiments 6 to 8 new-born mice (P0 – P5) were decapitated and the trigeminal ganglia ipsilateral to the site of inoculation were excised under a binocular. The ganglia were washed in phosphate buffered saline solution (PBS, Invitrogen) and collected in cold Leibowitz Medium (L15, Invitrogen). Ganglia were cut into small pieces and incubated (37°C, 95% air, 5% CO_2_) for 45 min in warm Dulbecco's modified essential medium (DMEM) containing 0.025% collagenase (type IA, Sigma).

Tissue was gently titrurated with a fire-polished glass pipette and the suspension was centrifugated at 200 g for 8 min. The obtained pellet was resuspended in culture medium with the following composition: DMEM/F-12 (1:1) with Glutamax (Invitrogen) supplemented with 10% fetal calf serum (Invitrogen), 100 μg/ml penicillin/streptomycin and 400 μg/ml Foscarnet (FO).

For calcium imaging experiments and patch-clamp recordings cells were plated on glass coverslips (Hecht, Germany) coated with poly-L-lysine (Sigma) (0.01%) and placed into petri dishes (Falcon, BD Biosciences, Heidelberg, Germany). Cells were kept in a humidified atmosphere (37°C, 5% CO_2_). One hour after plating, 2 ml culture medium was added to each dish. Neurons were grown for 16 h up to 4 days before being used.

Long-term maintenance of neurons in culture (several days to weeks) and presence of NGF has been shown to significantly alter phenotype of neurons, particularly with respect to TTX and capsaicin sensitivity [53-55]. Therefore, we maintained the neurons in our study for the shortest period possible to keep the experiments close to *in vivo *conditions. Most recordings were made within 24 h of preparation (except the data shown in Tab. 1). Cells were cultured without addition of NGF to cell medium.

### Multiphoton laser-scanning microscopy and imaging of intracellular calcium levels

Two-photon fluorescence within whole mount preparations of ganglia was excited by a mode-locked Ti:Sapphire laser (Mai Tai, 100 fs, 80 MHz; pumped by a 5-W CW laser; 860 nm; Spectra Physics; Germany) coupled to a laser scanning microscope (LSM510 Meta, Zeiss), fitted with a ×40 objective lens (Zeiss). A dichroic mirror (HFT KP 650) was inserted to the back aperture of the objective to reflect emitted light through detection optics and an emission filter (BP 500–550 IR) onto the photomultiplier. Image acquisition was controlled by custom software (Carl Zeiss AIM).

To study stimulus-induced changes of intracellular calcium, cells were loaded for 30 min with 2 μM Fluo-4 AM (Molecular Probes) in culture medium (Gibco, F-12) at 37°C. Cells were placed in an imaging chamber (10 × 5 × 1 mm) and continuously perfused (450 – 500 μl/min). Experiments were performed in an assay buffer containing (in mM) 140 NaCl, 5 KCl, 1 MgCl_2_, 2 CaCl_2_, 10 glucose, 10 HEPES. Drugs were applied in the assay buffer and could transiently superfuse the cells. Using the laser scanning microscope, emission intensity was measured for 10 min, every 200 – 400 ms using laser-excitation at 488 nm and an emission detection range from 504 to 536 nm. Stimuli (30 μM ATP, 45 mM KCl) were added after 10 sec.

### Electrophysiological recordings

Recordings were performed using the whole-cell mode of the patch-clamp technique. Cells were maintained in an extracellular recording solution equivalent to the assay buffer used in calcium imaging. Patch electrodes were pulled from borosilicate glass (1.2 mm O.D. × 1.17 mm I.D., Harvard apparatus, Edenbridge, Kent, UK) and fire polished to 6–8 MΩ tip resistance using a horizontal pipette puller (Zeitz Instr., Munich, Germany). The pipette solution contained (in mM) 140 KCl, 1 MgCl_2_, 0.1 CaCl_2_, 5 EGTA, 10 HEPES and 2 ATP, 0.1 GTP, 2 phosphocreatin, pH 7.4 for recordings from trigeminal neurons. Patch-clamp recordings were carried out at room temperature using a HEKA EPC9 amplifier. Adjustment to the capacity and series resistance was made by using the build in compensation algorithm of the amplifier. Membrane potential was held at -60 mV. Data were acquired using Pulse software. Capsaicin, menthol, α,β-meATP, ATP, and GABA were applied in the assay buffer and could transiently (4 s) superfuse the cells. For analyzing the TTX-resistance of sodium currents the patch-clamp recording protocol was applied before and 15 s after superfusing the cells with TTX (500 nM)-containing assay buffer.

### IB4 staining of TGNs in cell culture

For IB4-staining neurons were incubated (RT) with the plant lectin, IB4 conjugated to Alexa Fluor 488 (2 μg/ml) (Molecular Probes) for 15 min and then rinsed for 2 min. Alexa Fluor 488-staining was visualized with standard FITC filters (excitation 450 – 490 nm; emission filter LP 520). Neurons were considered IB4 positive if they had a continuous green rim around the perimeter at 25 × magnification.

### Tissue processing

At various time-points after infection, animals were sacrificed by decapitation and the brain was removed from the skull. Ganglia remained in the base of the skull and were placed in X-gal (5-bromo-4-chloro-3-indolyl-β-D-galactopyranoside) buffer (1 mg of X-gal per ml, 10 mM potassium ferrocyanide, 10 mM potassium ferricyanide, 2 mM MgCl_2_, 0.01% sodium deoxycholate, and 0.02% Nonidet P-40 in 0.1 M phosphate buffer, pH 7.4) for 1.5 h at 37°C. Tissue was stored in 4% paraformaldehyde in 0.1 M phosphate buffered saline (PBS, pH 7.4) at 4°C until photographed.

For cryosections of nasal epithelia and the orbital skin (inoculation sites) animals were sacrificed by decapitation and frontal parts of the heads were placed overnight in 4% paraformaldehyde in 0.1 M phosphate buffered saline (PBS, pH.7.4) at 4°C. This was followed by rinsing in 0.1 M PBS and cryoprotection with 30% sucrose in 0.1 M PBS overnight. Tissues were then frozen, sectioned on a cryostat (20 μm) and air dried for 30 min.

### Statistical analysis

Statistical significance (*: p < 0.05; **: p < 0.01) was assessed by Student's t-test or χ^2^-test. For statistical calculation SPSS and WinStat were used. "±" following numbers of values or percentages, indicates the standard deviation.

### Abbreviations

ATP: Adenosine triphosphate; β-gal: β-galactosidase; c: Control neurons; CMV: Cytomegalovirus; DRG: Dorsal root ganglion; FO: foscarnet; FRET: Fluorescence resonance energy transfer; HSV: Herpes simplex virus; TG: Trigeminal ganglion (*ganglion gasseri*); gG: Glycoprotein G; GFP: Green fluorescent protein; hpi: Hours post infection; IE: Immediate early; iv: *In vitro *infected neurons; mRFP1: Monomeric red fluorescent protein 1; PFU: Plaque forming units; PNS: Peripheral nervous system; PrV: Pseudorabies virus; TGN: Trigeminal neuron; Na_v_: Voltage gated sodium channels; YC2.1: Yellow Cameleon 2.1; X-gal: 5-bromo-4-chloro-3-indolyl-β-D-galactopyranoside

## Authors' contributions

ND carried out most of the experiments in this study and drafted the manuscript. MR contributed substantially to the electrophysiological recordings. BGK and TCM developed the PrV-Cam strain and gave advice in handling of viruses. HH participated in the design and coordination of the study. CHW conceived of the study, and helped to draft the manuscript. All authors read and approved the final manuscript.

**Table 1:** Electrophysiological analysis of infected neurons. Electrophysiological characterization of traced nasal, cutaneous, *in vitro *infected (*iv *infected), and uninfected (control) TGNs revealed no significant differences. APs: Action potentials

	control TGNs	*iv *infected TGNs	nasal TGNs	cutaneous TGNs	control TGNs	*iv *infected TGNs	nasal TGNs	cutaneous TGNs
	*Membrane potential/mV*				*Width of AP at 75 % of amplitude/ms*			
**n**	111	78	20	17	94	42	14	13
**mean**	-56,0	-54,3	-55,0	-54,4	2,27	2,26	1,96	2,40
**SD**	8,1	10,6	9,7	6,9	0,96	1,19	0,86	0,95
**SEM**	0,8	1,2	2,2	1,7	0,10	0,18	0,23	0,26
**range**	38	66	33	23	4,60	5,40	3,00	3,40
								
	*Threshold for activation of VGSCs/mV*				*Maximal amplitude of APs due to current injection/mV*			
**n**	106	45	12	14	95	42	14	14
**mean**	-32,9	-32,5	-33,0	-31,5	41,3	36,8	37,9	51,3
**SD**	9,6	11,1	11,3	12,2	16,3	15,3	15,3	10,3
**SEM**	0,9	1,7	3,3	3,3	1,7	2,4	4,1	2,8
**range**	60	60	40	36	73	69	48	36,0
								
	*Maximal amplitude (overshoot) of APs/nA*				*Ih current (-130 mV)/pA*			
**n**	12	10	16	14	67	14	7	10
**mean**	-3,80	-5,22	-3,78	-7,2	-258	-226	-327	-130
**SD**	1,95	2,74	2,69	3,21	221	205	325	109
**SEM**	0,56	0,87	0,67	0,86	27	55	123	35
**range**	6,25	7,49	9,24	12,07	1066	639	845	363
								
	*Threshold of current injection for eliciting APs/pA*				*Amplitude of "sag"/mV*			
**n**	95	42	14	n.d.	11	18	8	3
**mean**	258	237	213	n.d.	10,1	18,6	22,6	17,0
**SD**	246	201	202	n.d.	6,2	10,5	15,6	8,0
**SEM**	25	31	54	n.d.	1,9	2,5	5,5	4,6
**range**	990	800	770	n.d.	17	37	44	16,0
								
	*Membrane potential due to current injection eliciting APs/mV*				*Threshold of current injection for Ih-activation/mV*			
**n**	94	42	14	14	11	18	8	3
**mean**	-30,1	-31,5	-34,6	-32,9	-79,5	-83,4	-81,9	-87,3
**SD**	8,1	10,5	7,9	7,8	7,6	20,4	10,3	7,5
**SEM**	0,8	1,6	2,1	2,1	2,3	4,8	3,7	4,3
**range**	54	46	29	27	26	83	31	15
